# Protective behaviors during COVID-19 confinement measures in Greece: the role of anxiety, perceived risk and risky-choice framing

**DOI:** 10.3934/publichealth.2023021

**Published:** 2023-04-25

**Authors:** George Koulierakis, Anastasia Dermatis, Dimitris Zavras, Elpida Pavi

**Affiliations:** 1 Department of Public Health Policy, School of Public Health, University of West Attica, Athens, Greece; 2 Laboratory for Health Technology Assessment, Division of Health Systems and Policy. Department of Public Health Policy, School of Public Health, University of West Attica, Athens, Greece

**Keywords:** protective behaviors, anxiety, risk perception, risky-choice framing, COVID-19

## Abstract

Confinement measures at the beginning of the COVID-19 pandemic imposed major changes on the global population. The aim of this study was to explore the level to which the public adhered to protective guidelines by adopting the most appropriate behaviors at that time (such as hand washing with soap and using sanitizer gel) and to identify the determinants of these behaviors. A purposive sample of 1013 individuals was invited and voluntarily participated in the online survey. The questionnaire collected information on demographic data, hand washing, risk perception, anxiety (through the S = Anxiety scale of STAI) and risky-choice framing. Results showed increased levels of anxiety, a moderate perception of the risk of catching coronavirus and increased adoption of protective behaviors, such as handwashing and cleaning surfaces with disinfectant/antiseptic products. Multiple ordinal logistic regression models showed that being female, more educated and cleaning home with disinfectant / antiseptic products predicted handwashing with soap. Additionally, having an increased perception of getting the coronavirus, being older and cleaning the home with disinfectant / antiseptic products predicted handwashing with antiseptics. Public health interventions should take into consideration the unified cleaning pattern and the combined effect of sociodemographic variables and risk perception on the adoption of protective behaviour in the context of a health crisis which is out of people's control.

## 1. Introduction

The SARS-COV-2 pandemic has been an acute public health threat [1]. It started in January 2020, when the World Health Organization declared the novel acute respiratory syndrome coronavirus (2019-nCoV) to be a Public Health Emergency of international concern [2]. As of September 28, 2022, 613,410,706 confirmed COVID-19 cases, including 6,518,749 deaths, have been reported globally [3]. In Greece, the first confirmed COVID-19 case was reported on February 26, 2020, while the first COVID-19-related death was reported on March 12, 2020 [4]. Within two months – up to April 27, 2020, 136 COVID-19-related deaths, among 2,534 laboratory confirmed cases, were recorded [5]. As of September 25, 2022, a total of 4,920,192 cases and 33,111 deaths have been recorded in Greece [6].

In order to restrict the number of infections and protect individuals, numerous precautionary measures have been implemented around the world since March 2020. During that period, all countries adopted different policies concerning quarantine measures and day-to-day restrictions, while the effectiveness of those policies was assessed to inform governments in responding to future pandemics [7]. In Greece, on February 27, 2020, the government cancelled all carnival celebrations. From March 10, 2020, gradual lockdown decisions were issued concerning all educational institutes, bars, cafes, restaurants, shopping malls, museums, stadiums and athletic settings, retail shops and religious gatherings, until March 23, 2020 when strict measures on the general population's movement were taken, imposing penalties for breach. All measures aimed at limiting down non-essential movements in an attempt to achieve physical (social) distancing. In parallel, an intensive “Staying at Home” campaign at the national level was implemented. Alongside the measures, recommendations were also made for individual lifestyle changes, by restricting social, work or school contacts and travel. In Greece, the vast majority of the general population complied with the measures and consequently, Greece's death toll was low. Nevertheless, it has been estimated that the impacts of the outbreak were, as everywhere worldwide, multifaceted and affected many parts of the economic, social and political life of the country [8].

The long periods of quarantine lead to a significant financial and psychological burden, such as increased levels of anger, confusion and stress, as well as extensive behavioral changes worldwide [9]–[16]. In Portugal, empirical evidence showed that a large amount of the population experienced phobias, fear and anxiety linked to COVID-19, influenced by the perceived risk of contracting COVID-19. The higher the risk of contracting the disease, the higher the phobias, fear and anxiety [17]. A different study in Portugal indicated that individuals expressed fear and anxiety due to COVID-19, related to concerns for tourism, hospitality and travel, as they considered that COVID-19 would influence their leisure time and holidays [18]. At the same time, initiatives to alleviate the burden were adopted [19]. In Greece, previous studies indicated that the public experienced high levels (35.7%) of COVID-19-related fear, moderate to severe (22.8%) depressive symptoms, as well as moderate to severe (77.4%) anxiety symptoms [20]. Increased levels of depression, anxiety, stress and negative affect were found in another study among students, while life disruption, perceived risk of the disease and female gender were risk factors for mental health problems [21]. Additionally, a recent study in Greece demonstrated a strong association of COVID-19 fear with increased levels of health stress, anxiety, and posttraumatic stress among the population [22].

During the preliminary phase of the pandemic, mass media announced daily COVID-19 mortality and morbidity rates [23]. The exposure to mortality salience and the conscious conception of dying led to an increase in self-protective behaviors such as sanitizing or handwashing, in order to minimize the risk of contracting the deadly virus [24], although findings were not always consistent [25]. Despite the fact that adhering to government health policies was crucial, reports showed that individuals' preventive behaviors were insufficient [26],[27]. In Greece, research indicated that 60% of the general population did not have appropriate hand hygiene behavior [28]. Additionally, another study showed that frequent or constant handwashing was reported by 56.7% and 29.8% respectively, while only 16.7% indicated constant use of face masks. Attitudinal and socioeconomic factors critically affected the public's engagement in these preventive behaviors [29].

Evidently, adherence to public health policies mainly depends on people's perception of COVID-19 and the risks associated with it. Perceived risk is defined as “an individual's subjective evaluation of their risk of a disease or an unfavorable outcome, often linked with performing a certain risky behavior” [30]. Recent research suggests that individuals who feel that they will be harmed by the disease and are at high risk of getting ill show high adaptation to preventive behaviors [31],[32]. Furthermore, numerous studies have indicated that state anxiety is a psychological characteristic which influences the success of public health policies during pandemics, notably hygiene practices and risk communication [33]. Anxiety is a mental state characterized by an intense sense of tension, worry or apprehension, relative to something adverse that might happen in the future. It is distinguished to “state anxiety” defined as a temporary reaction to adverse events, and “trait anxiety”, which is a more stable personality feature [34]. Cognitive behavioral models support that anxiety appears across a continuum, ranging from low to high levels. Low levels of state anxiety have been associated with the notion that an individual is at low risk of contracting the virus, whereas high levels of state anxiety have been associated with the notion that the individual is at high risk of getting ill [35],[36]. Research during the H1N1 pandemic showed that individuals with low levels of anxiety perceived themselves as maintaining a low risk of contracting the disease, thus washed their hands less frequently than those with high levels of anxiety who viewed themselves as being at high risk [37].

Over several decades, scientists have been interested in the fact that individuals reply differently to contrasting but objectively analogous interpretations of the same problem. Risky-choice framing has been researched by Tversky & Kahneman [38] in their academic work on the Asian Disease Problem (ADP). The ADP incorporates two scenarios, that is either positively (gain) or negatively (loss) framed. Individuals are presented with two choices when 600 people are facing imminent death: In the gain frame, choice A is that 200 individuals will be saved, and choice B consists of a 1/3 probability that all 600 individuals will be saved, with a 2/3 probability that no individuals will be saved. In the loss frame, choice C is that 400 individuals will die and choice D consists of a 1/3 probability that no one will die, with a 2/3 probability that 600 will die. When this problem was introduced in a way that emphasized individuals being saved (framed positively), empirical evidence showed that 71% of the individuals chose the A option. On the contrary, when the problem was introduced in a way that emphasized on those who would lose their life (framed negatively), 78% of the individuals chose the D option [38]. Extended research showed that not only lay individuals but also individuals in the medical profession are vulnerable to this framing effect [39].

Under such circumstances, the COVID-19 pandemic provided the opportunity to test, in a real-life setting, theories concerning the interactive outcome of framing and situational factors on decision making and risk appraisal [40],[41]. The notion of decision making has been studied to a great extent, incorporating how risky situations influence our decisions and how various circumstances modify the way the risk is evaluated. Acknowledging how emotions guide the influence of problem framing on individuals, decisions can have actual implications. Analogies to pandemic administration, investigating whether choice under risk is associated with complying with healthcare regulations could provide valuable insight into the possibility of which is the framing that can be used to custom messages regarding particular groups of individuals [42].

Thus, the aim of this study was to explore the level that the general public in Greece - during the early phase of the pandemic - adhered to protective guidelines by adopting the most appropriate behaviors at that time (hand washing with soap and using sanitizer gel) and identify potential cognitive and psychosocial determinants of these behaviors.

## 2. Materials and methods

### 2.1. Study design

A cross-sectional on-line survey (Exposome@Home|COVID-19 - Greece) was designed and conducted among a sample of the general adult population (≥18 years, who live for at least 1 year in Greece), during the implementation of the physical distancing measures in Greece (May – June 2020). The study was conducted by the Department of Public Health Policy of the School of Public Health of the University of West Attica, in collaboration with the Cyprus International Institute for Environmental and Public Health, Cyprus University of Technology.

#### 2.1.1. Sample

Given the lack of knowledge on similar studies due to the uniqueness of the pandemic emergency and the measures proposed or taken globally, it was not possible to use prior knowledge for sample size estimation. However, a target sample size of 1000 participants/filled questionnaires was expected to provide high coverage of the general Greek population, based on previous experience with the National Surveys “Health and Well-being” which the Department of Health Economics of the former National School of Public Health carried out at regular intervals. A stratified quota sample of the adult population of Greece, based on age, gender and region of residence, was predetermined with a targeted total of 1000 subjects. Eventually, 1013 individuals answered the on-line questionnaire voluntarily participating in the study.

#### 2.1.2. Procedure

By employing a market research company, an effective communication strategy was designed for the dissemination of the link of the questionnaire: it was posted on the “Announcements/News” site of the Department of Public Health Policy at the University of West Attica, and was promoted through social media and the internet (Facebook, Twitter, Google) by using legal marketing engines in order to reach the targeted adult population quota profile.

### 2.2. Data collection

#### 2.2.1. Demographic variables

Basic sociodemographic data was recorded as follows: gender (1 = male, 2 = female); age; financial affordability was assessed through replies to the question “Considering your total family income and your liabilities, which of the following phrases best suits your financial situation at this period?” (1 = “We make do very well”, 2 = “We make do quite well”, 3 = “We make do well”, 4 = “We have some financial difficulties”, 5 = “We have quite a lot of financial difficulties”, 6 = “We have very serious financial difficulties”); marital status (1 = “Not married”, 2 = “living with partner”, 3 = “Married”, 4 = “Widowed (not re-married)”, 5 = “Divorced” (never re-married”), educational level and employment status. Self-reported health was rated on a five-point ordinal scale (1 = “very bad”, 2 = “bad”, 3 = “fair”, 4 = “good”, 5 = “very good”, 6 = “Not sure/don't remember”) in response to the question “How would you rate your health today?”.

#### 2.2.2. Anxiety measure

The level of anxiety was measured with the S-Anxiety scale of the State-Trait Anxiety Inventory (STAI form-Y-1) [43], as it was validated in Greek [44]. The scale consists of twenty statements that evaluate how the respondent feels “right now, at this moment”, at a 4-poin scale: (1) not at all, (2) somewhat, (3) moderately, (4) very much so. Higher scores represent higher levels of anxiety. A cutoff score of 40 is commonly used to define probable clinical levels of anxiety. Cronbach's alpha was 0.94 for the scale, indicating very good reliability.

#### 2.2.3. Risk perception

Participants' perception of risk was assessed through replies to the question “How likely do you think you are, you personally, to get infected by the coronavirus?” (1 = “Extremely unlikely”, 2 = “Rather unlikely”, 3 = “Neither unlikely, nor likely”, 4 = “Rather likely”, 5 = “Almost certain”, 98 = “Do not know”, 99 = “Not answer”)

#### 2.2.4. Framing effect

The positive framing of the Asian Disease Problem, adapted to COVID-19 was used as a measure of the risky-choice framing effect. Participants were presented with the following statement: “To address the spread of coronavirus, between the following two hypothetical scenarios of measures, which would you propose to be adopted to prevent more infections?” (1 = “Measures that will definitively save 2,000 people”, 2 = “Measures by which there is 1/3 chance of saving 6000 people and 2/3 chance of saving no one”, 98 = “Do not know”, 99 = “Not answer”).

#### 2.2.5. Protective behaviors

Information regarding protective behaviors were obtained by asking participants to report the frequency of adopting one of the following measures: i) washing hands with soap, ii) washing hands with antiseptic products, coded as “Never”, “1–3 times a day”, “4–7 times a day”, “>7 times a day”. Additionally, participants were asked to report the average number of days per week that they were using disinfectant/antiseptic products to clean the sanitary facilities (e.g. bathrooms, toilets), kitchen surfaces, or other places and to mop the floors. Participants were also asked to mention previous preventive behaviors, namely vaccination for influenza (ever) and pneumococcus (during the last 5 years).

### 2.3. Ethical issues

Participation in the study was completely voluntary. Participants were informed about their right not to participate and/or withdraw from the study at any point, without their responses to be kept. Informed consent to participate was provided by clicking the relevant button at the end of the 1^st^ Screen of the on-line questionnaire, where all relevant study information was provided. Respondents were also able to address questions for more information (special e-mail address and telephone number were also provided on the 1^st^ Screen), as well as submit complaints to a staff member of the Department who was involved to the survey (name and e-mail address were given on 1^st^ Screen). The protocol of the study was approved by the Research Ethics Committee of the University of West Attica (Decision No. 30548/05–05–2020).

### 2.4. Statistical analysis

A descriptive analysis to summarize participants' demographics was applied, using percentages for categorical variables and means and standard deviations for continuous variables and counts. Similarly, we analyzed respondents' COVID-19 related protective behaviors during the confinement measures, their level of anxiety and their perceptions of risk catching the virus. Ordinal logistic regression models were fitted to evaluate the influence of demographic and socioeconomic characteristics, anxiety, perceived risk, risky-choice framing and past behaviors on the reported protective behaviors (hand washing with soap and antiseptics) during the confinement measures. The proportional odds assumption was tested through the Brant test [45]. The models' goodness of fit was tested through the Lipsitz et al. test [46]. In addition, the models were tested for specification error through the link test. The analysis was performed with the STATA 17 statistical software package. Specifically, the commands ologit, brant [47], ologit, gof [48] and linktest were used. All statistical tests were two-sided at 5% significance level.

## 3. Results

Participants' mean age was 48.6 years (SD = 14.9 years). Table 1 shows the basic sociodemographic characteristics of the sample. As presented in Table 1, participants were almost equally divided to males and females, the majority (46.2%) were married, and almost one out of five (24.2%) had completed secondary education. Of the sample, 359 individuals (35.4%) were employed in the private sector, either on a full-time or a part-time basis. More than half of participants (53.2%) reported that they faced financial difficulties to come up with their liabilities.

**Table 1.** Participants' basic demographic characteristics.

	**n**	**%**
**Gender**		
Male	514	50.7
Female	499	49.3
**Marital status**		
Not married	315	31.1
Living with partner	80	7.9
Married	468	46.2
Widowed (not re-married)	47	4.6
Divorced (never re-married	103	10.2
**Educational level**		
Never been at school /Not completed primary education	4	0.4
Primary education	26	2.6
Completed secondary education	245	24.2
Post-secondary education	83	8.2
Higher technical education (tertiary)	127	12.5
University	263	26
Μaster's degree	204	20.1
Ph.D. degree	61	6
**Employment status**		
Employed (public sector)	189	18.7
Employed (private sector)	359	35.4
Unemployed	152	15
Household (no payment)	33	3.3
Retired	166	16.4
Student	71	7
Army service	5	0.5
Other	38	3.8
**Financial affordability**		
We make do very well	58	5.7
We make do quite well	122	12.1
We make do well	275	27.3
We have some financial difficulties	286	28.3
We have quite a lot financial difficulties	160	15.9
We have very serious financial difficulties	91	9

Table 2 presents participants' health status, their perception of the risk of getting COVID-19, and their protective behaviors during the confinement measures. As shown in Table 2, the vast majority of the participants (84.7%) reported a good/very good status of their health. Anxiety levels were high, as the average score of S-Anxiety scale was above the cutoff point of 40, thus indicating potential clinical levels of anxiety. Most of participants (45.6%) were seemed to be risk-averse, as they chose the secure option of the ADP. Almost half of them (49.6%) appeared to be balanced as far as their perception regarding the risk of getting COVID-19, while almost equal percentages reported limited (22.6%) and increased likelihood (23.4%). The majority of respondents (55.1%) had never been vaccinated for influenza, as had an even higher percentage (74.1%) for pneumococcus during the last 5 years. As far as protective behaviors, the majority claimed frequent (“4-7 times a day”: 39.9%) or excessive (“>7 times a day”: 28.5%) handwashing with soap, while only 3.3% mentioned that they never washed their hands. As far as washing hands with antiseptic products, the majority (38.1%) mentioned constant (“1-3 times a day”) practice, while a notable percentage (24.7%) had never used antiseptics to wash their hands. Finally, participants reported an increased average of 4.8 (SD = 2.5) days per week that cleaned their kitchen surfaces with antiseptic products.

**Table 2.** Health status, perceived risk, framing and protective behaviors of the study participants.

	**N (%)**	**Mean**	**SD**
**Self-reported health status**			
Very bad	5 (0.5)		
Bad	20 (2)		
Fair	128 (12.6)		
Good	407 (40.2)		
Very good	451 (44.5)		
Not sure/don't remember	2 (0.2)		
**State Anxiety**		44.7	13.7
**Risky-choice framing**			
Measures that will definitively save 2000 people	462 (45.6)		
Measures by which there is 1/3 chance of saving 6000 people and 2/3 chance of saving no one	115 (11.4)		
Do not know	325 (32.1)		
Not answer	111 (11)		
**Perceived risk of getting infected**			
Extremely unlikely	89 (8.8)		
Rather unlikely	140 (13.8)		
Neither unlikely, nor likely	502 (49.6)		
Rather likely	212 (20.9)		
Almost certain	25 (2.5)		
Do not know	43 (4.2)		
Not answer	2 (0.2)		
**Ever vaccinated for influenza**			
YES	390 (38.5)		
NO	558 (55.1)		
Do not know	60 (5.9)		
Not answer	5 (0.5)		
**Pneumococcus vaccination during the last 5 years**			
YES	190 (18.8)		
NO	751 (74.1)		
Do not know	71 (7)		
Not answer	1 (0.1)		
**Washing hands with soap**			
Never	33 (3.3)		
1−3 times a day	287 (28.3)		
4−7 times a day	404 (39.9)		
>7 times a day	289 (28.5)		
**Washing hands with antiseptic products**			
Never	250 (24.7)		
1−3 times a day	386 (38.1)		
4−7 times a day	232 (22.9)		
>7 times a day	145 (14.3)		
**Use disinfectant/antiseptic products to... (days/week)**			
Clean bathrooms, toilets		3.60	2.5
Clean kitchen surfaces		4.28	2.5
Clean other places		2.69	2.2
Mop the floors		2.65	2.1

**Table 3.** Ordinal Logistic regression of hand-washing with soap and antiseptic products.

	**Model 1 - Hand-washing (soap)**			
**Variables**	**OR**	**(95% CI)**	**SE**	**P**
Gender	0.46	0.36−0.58	0.05	0.000
Use disinfectant/antiseptic products to clean	1.28	1.21−1.36	0.04	0.000
Educational level	1.14	1.07−1.22	0.03	0.000
	**Model 2 - Hand-washing (antiseptics)**			
Risk perception	1.37	1.20−1.56	0.09	0.000
Use disinfectant/antiseptic products to clean	1.45	1.36−1.55	0.04	0.000
Age	1.01	1.00−1.02	0.004	0.000

According to the link tests (Table 4), both models do not suffer from specification errors.

**Table 4.** Link test. Hand-washing with soap and antiseptic products.

	**Model 1 - Hand-washing (soap)**		
**Variables**	**coefficient**	**P**	**(95% CI)**
h	0.96	0.001	0.373–1.562
h^2^	0.01	0.912	−0.191–0.214
	**Model 2 - Hand-washing (antiseptics)**		
h	0.980	0.018	0.167–1.793
h^2^	0.003	0.962	−0.142–0.149

The Brant tests indicated that the assumption of proportional odds is not violated: [model 1: p_(all)_ = 0.143, p_(use disinfectant products)_ = 0.079, p_(gender)_ = 0.067, p_(educational level)_ = 0.663], [model 2: p_(all)_ = 0.229, p_(perceived risk)_ = 0.244, p_(use disinfectant products)_ = 0.449, p_(age)_ = 0.097]. The Lipsitz tests indicated that both models had a good fit (model 1: p = 0.34, model 2: p = 0.30).

## 4. Discussion

The current study investigated the extent to which the general public in Greece adhered to protective guidelines by practicing the most appropriate health behaviors during the COVID-19 pandemic, namely hand washing with soap and sanitizer gel. Additionally, this study aimed to identify potential cognitive and psychosocial determinants of these behaviors. It has been widely documented that the SAR-COV-2 pandemic had a significant impact on public health [1]. Countries around the world adopted preventive measures in order to limit the number of infections, and to protect individuals from the deadly virus. Since March 2020, strict government measures have been enforced globally, regarding quarantine protocols and daily restrictions [7], intended to attain social distancing in order to reduce contamination [9]. In Greece, confinement measures were gradually posed since February 2020 when the Greek government canceled the traditional carnival festivities, followed by a strict lock-down regulations imposed on the general population on March 23, 2020.

During the long periods of social isolation, the general public experienced acute levels of psychological impact. Individuals reported increased levels of anxiety, depression, and fear related to COVID-19. Results of the current study indicated that during the primary quarantine phase of the COVID-19 pandemic, anxiety levels were high in Greece (the average S-Anxiety scale score was 44.7, that is over the cutoff point of 40), approaching clinical levels. Thus, our findings confirm previous studies in Greece, which showed that during the long periods of social isolation, the general public experienced increased levels of anxiety, depression and fear related to COVID-19 [20]–[22]. Our results are also compatible with studies showing that the prolonged phase of quarantine led to significant psychological effects in different countries worldwide [9],[10],[15],[17],[18].

As for how risk perception framing affects people's decision making, our results showed that 45.6% of the participants were opposed to risk and chose the secure option when faced with the Asian Disease Problem, thus confirming findings by Rachev et al. who suggested that high risk aversion is associated with elevated levels of anxiety [42]. Further scientific evidence indicates that increased levels of anxiety are linked to people's disposition to refrain from risky decision making [49]. Moreover, our results are in line findings reporting that anxiety and distress have a significant effect on decision making than other determinants, such as income [50].

As indicated by scores on protective behaviors (handwashing with soap, handwashing with antiseptics), the majority of the participants in this study washed their hands with soap frequently (39.9%), or excessively (28.5%), whereas a high percentage of individuals (38.1%), washed their hands with antiseptic products. Additionally, gender, education level and cleaning the house with disinfectant/antiseptic products were the only determinants of handwashing with soap, while risk perception, cleaning the house using disinfectant/antiseptic products and age predicted handwashing with antiseptics. Previous research has suggested that emotional states (i.e. anxiety, fear) and perceived risk affected compliance with SAR-COV-2 safety recommendations, particularly hand washing [26],[51]. Although, in our study, we did not find anxiety as an independent predictor of hand washing, our findings seemed to confirm the interrelations between risk perception, anxiety and worry, as the most critical determinants of preventive behavior [24]. It seems that people's sense of control could be used as a potent explanation to interpret our data: as people faced at that time a completely unknown and out of their control situation, they adopted the only available measure for their protection [51].

The role of sociodemographic characteristics such as age, gender and educational level in adopting preventive behaviors and/or adherence to government preventive policies appears constantly in the literature, either as individual associates [29],[52],[53], or in relation to perceived risk [54]. In our study, older individuals, women and those with higher education adopted COVID-19 related protective behaviors more frequently. Evidence has indicated that individuals aged 65 and above are at risk of acute health issues from COVID-19 and death rates among the senior population have been heightened by complications due to the virus [55]. Our findings regarding age are consistent with those of Brankston et al., who documented that perceived risk increased adherence towards preventive behaviors for older individuals [56]. As for gender, women in our study had better adherence towards protective health policies than men. This could be due to gender stereotypes [57] and the fact that women take fewer risks than men [58]. Finally, a strong determinant for adopting frequent COVID-19 protective behaviors in our study was education. Individuals with higher education washed their hands with soap more frequently than those with lower education, thus confirming previous research and is consistent with evidence by Wise et al., indicating that participants with college-level education had greater adherence towards preventive behaviors [59].

At the same time, participants in our study, seemed to adopt a unified protective behavioral pattern: those who used disinfectant /antiseptic products to clean their houses adopted a more frequent hand washing [60].

The current study investigated a purposive sample of the Greek general public and was based on a potent methodological design. Although this type of methodology was common at that period onwards and forced by the strict limitations, our research has some limitations. Due to the fact that our on-line survey was structured on self-reported ratings, this could trigger social desirability bias. Furthermore, the cross-sectional nature of our survey prevents the development of casual inferences. Moreover, in order to recruit our participants, a snowball sampling method was used, which means we are not guaranteed a national sample. Thus, generalization cannot be securely assumed.

## 5. Conclusions

Our findings, based on 1013 participants, support significant implications for both practice and theory. High levels of anxiety made participants avoid risk averse choices, as shown in the Asian Disease Problem. Furthermore, perceived risk and other demographic characteristics influenced the adherence to preventive behaviors during the initial COVID-19 quarantine phase in Greece. Hand washing with soap and hand washing with antiseptic products was high during this phase. These behaviors did not seem to be connected with previous protective public health practices like influenza and pneumococcus vaccination, which were reported as low, indicating the unique characteristics of the COVID-19 pandemic. Further research should be conducted on the fundamental processes through which COVID-19 mortality and morbidity approaches may influence protective behaviors. Such processes may involve people's unconscious operations, behavioral intentions and understanding of COVID-19 transmission. Knowing the cognitive and psychosocial determinants of protective behaviors during the COVID-19 pandemic could help protect the general public, develop efficient preventive actions and target communication strategies in order to vigorously participate in the fight against the deadly virus.
